# Adverse Outcomes Following Exposure to Perfluorooctanesulfonamide (PFOSA) in Larval Zebrafish (*Danio rerio*): A Neurotoxic and Behavioral Perspective

**DOI:** 10.3390/toxics12100723

**Published:** 2024-10-04

**Authors:** Nikita David, Emma Ivantsova, Isaac Konig, Cole D. English, Lev Avidan, Mark Kreychman, Mario L. Rivera, Camilo Escobar, Eliana Maira Agostini Valle, Amany Sultan, Christopher J. Martyniuk

**Affiliations:** 1 Center for Environmental and Human Toxicology, Department of Physiological Sciences, College of Veterinary Medicine, University of Florida, Gainesville, FL 32611, USA; nikitadavid@ufl.edu (N.D.); eivantsova@ufl.edu (E.I.); isaac.konig@ufla.br (I.K.); coleenglish@ufl.edu (C.D.E.); lavidan@ufl.edu (L.A.); mkreychman@ufl.edu (M.K.); mario.rivera@ufl.edu (M.L.R.); camilo.escobar@ufl.edu (C.E.); emavalle@unifesp.br (E.M.A.V.); amanysultan2025@gmail.com (A.S.); 2 Department of Chemistry, Federal University of Lavras (UFLA), Minas Gerais, Lavras 37203-202, Brazil; 3 Instituto de Ciencias Ambientais, Quimicas e Farmaceuticas, Universidade Federal de São Paulo, Campus Diadema, Diadema 09972-270, Brazil; 4 Animal Health Research Institute, Agriculture Research Centre, Giza 3751254, Egypt; 5 UF Genetics Institute, Interdisciplinary Program in Biomedical Sciences Neuroscience, University of Florida, Gainesville, FL 32611, USA

**Keywords:** perfluorinated chemicals, aquatic toxicology, neurotoxicity, behavior

## Abstract

Toxicity mechanisms of per- and polyfluoroalkyl substances (PFASs), a chemical class present in diverse ecosystems, as well as many of their precursors, have been increasingly characterized in aquatic species. Perfluorooctanesulfonamide (PFOSA, C_8_H_2_F_17_NO_2_S) is a common precursor of perfluorooctane sulfonic acid (PFOS), a long-chain PFAS. Here, we assessed sub-lethal endpoints related to development, oxidative stress, transcript levels, and distance moved in zebrafish embryos and larvae following continuous exposure to PFOSA beginning at 6 h post-fertilization (hpf). PFOSA decreased survival in fish treated with 1 µg/L PFOSA; however, the effect was modest relative to the controls (difference of 10%). Exposure up to 10 µg/L PFOSA did not affect hatch rate, nor did it induce ROS in 7-day-old larvae fish. The activity of larval fish treated with 100 µg/L PFOSA was reduced relative to the solvent control. Transcripts related to oxidative stress response and apoptosis were measured and BCL2-associated X, apoptosis regulator (*bax*)*,* cytochrome c, somatic (*cycs*)*,* catalase (*cat*), superoxide dismutase 2 (*sod2*) were induced with high concentrations of PFOSA. Genes related to neurotoxicity were also measured and transcript levels of acetylcholinesterase (*ache*), elav-like RNA binding protein 3 (*elavl3*), growth-associated protein 43 (*gap43*), synapsin II (*syn2a*), and tubulin *3 (tubb3)* were all increased in larval fish with higher PFOSA exposure. These data improve our understanding of the potential sub-lethal toxicity of PFOSA in fish species.

## 1. Introduction

Since the 1930s, per- and polyfluoroalkyl substances (PFASs) have been used in a variety of consumer and commercial goods, including textiles, surfactants, and food packaging materials. These compounds comprise short or long carbon chains, where short-chain PFAS are considered less harmful than long-chain PFAS [1]. According to the Danish Environmental Protection Agency, because long-chain PFAS persist in the environment, their production usage has been gradually phased out since the early 2000s [2]. Perfluorooctanesulfonamide (PFOSA, C_8_H_2_F_17_NO_2_S) is a common precursor of perfluorooctane sulfonic acid (PFOS), a long-chain PFAS [3], and it is a synthetic compound used to produce non-stick, waterproof, and stain-repellent coatings [4]. Most studies to date focus on the presence and toxicity of other commonly used PFAS, such as PFOS or perfluorooctanoic acid (PFOA) [5,6,7]. Currently, toxicity data regarding the precursor PFOSA are lacking.

There are a few studies that report on the environmental presence of PFOSA. Konwick et al. (2008) found that PFOSA ranged from 162 to 283 ng/L in the Conasauga River, Georgia, United States [8]. Additionally, other studies reported the following ranges for PFOSA: 0.09–20,000 µg/kg in surface soil, 0.07–2500 µg/kg in subsurface soil, 15 µg/L in surface water, and 12 µg/L in groundwater across various testing sites worldwide [9,10]. In regards to PFOSA within fish tissues, Fair et al. (2019) measured different PFAS within edible fish species from South Carolina, United States [11]. In whole fish, the average relative percent of PFOSA in mullet, spot, croaker, red drum, and seatrout was 2.04, 3.81, 3.64, 3.12, and 4.66%, respectively, whereas, in fillets, the same species had an average relative percent of 1.44, 2.03, 0.70, 2.18, 4.65, and 1.79%, respectively. PFOSA was also found to range between 0.105 and 16.4 ng/mL in serum across various fish species, including crucian carp (*Carassius auratus*), tilapia (*Oreochromis niloticus*), common carp (*Cyprinus carpio*), and leather catfish (*Clarias lazera*) [12]. However, the mechanisms of uptake, metabolism, and toxicity of PFOSA are relatively unknown for aquatic species. One study reports that the half-life of PFOSA in rainbow trout (*Oncorhynchus mykiss*) is 6.0 ± 0.4 days following a 30-day dietary exposure to 10 µg/g wet weight PFOSA and a 30-day depurination period [13]. Thus, PFOSA is measurable in fish tissues and may pose a health risk to both aquatic/terrestrial animals and humans. 

According to studies, adverse morphological and physiological effects in aquatic organisms are potential consequences associated with the environmental presence of long-chain PFAS [14,15]. For example, studies show that PFOSA exerts cardiotoxicity in zebrafish (*Danio rerio*). Exposure to 0.1–100 µg/L PFOSA has been reported to reduce cardiac output, heart rate, stroke volume and reduce cardiac vasoconstriction-related genes [16]. PFOSA has also been reported to significantly increase sinus venosus and bulbus arteriosus distances at 10 and 100 µg/L [16,17]. Other studies report that exposure to PFOSA can induce hepatic and renal toxicity in zebrafish [18,19]; however, limited studies investigate the neurotoxic potential of PFOSA in developing fish. Consequently, the objectives of this study were to evaluate the neurotoxicity potential of PFOSA. To achieve this, we measured developmental endpoints, reactive oxygen species (ROS), locomotor behavior, and genes related to oxidative damage response, apoptosis, and neurotoxicity as indicators of central nervous system damage. We hypothesized that neurotoxicity endpoints would reflect dose response increased in PFOSA exposure, suggesting adverse effects on the nervous system.

## 2. Materials and Methods

### 2.1. Chemical Preparation

Perfluorooctanesulfonamide (PFOSA, (1,1,2,2,3,3,4,4,5,5,6,6,7,7,8,8,8-heptadecafluorooctane-1-sulfonamide Perfluorooctane sulfonamide) (CAS Number: 754-91-6, purity > 95%) was purchased from Fisher Scientific (Thermo Scientific Chemicals, Waltham, MA, USA, Cat# AC459640010). PFOSA stock solutions were prepared in dimethyl sulfoxide (DMSO, dimethyl sulfoxide, CAS 67-68-5, Sigma-Aldrich, Inc., St. Louis, MO, USA) and added to embryo rearing media (ERM) containing the zebrafish embryos. The ERM recipe can be located in Westerfield [20]. Exposure solutions were prepared daily to yield final nominal environmentally relevant concentrations of 0.1, 1, 10, and 100 µg/L PFOSA with a final concentration <0.1% *v/v* DMSO in experimental treatments.

### 2.2. Maintenance and Egg Production of Zebrafish

Zebrafish (AB x Tübingen, *Danio rerio*) husbandry has been described previously [21,22] and rearing and staging of zebrafish embryos followed that described previously [23]. The Supplemental Methods section contain full details on zebrafish husbandry and water quality. The University of Florida Institutional Animal Care and Use Committee approved all experiments (UF_IACUC202300000140).

### 2.3. PFOSA Exposure Regime

Fertilized and normally developing eggs were selected at ∼6 h post-fertilization (hpf) and were assigned into experimental groups in random fashion [ERM, 0.1% DMSO, or one dose of 0.1, 1, 10 or 100 µg/L PFOSA]. Four independent experiments were conducted. There were 5 to 6 replicate glass beakers for each treatment group containing between 20 and 30 embryos and 10 mL of ERM per beaker. Once chemicals were added, the beakers were placed inside an incubator at 27 ± 1 °C. Mortality, deformities, hatch times and images using an EVOS™ FL Auto Imaging System (ThermoFisher Scientific, USA) were collected daily. Deformity assessments included the presence of spinal lordosis and edema (yolk sack/pericardial) was noted each day. Water was renewed each day with a 90% water change using freshly made stocks of PFOSA.

### 2.4. Reactive Oxygen Species

Following fertilization in the morning, embryos at 6 h post fertilization were collected and treated as per above for 7 days in the ERM with designated concentrations of PFOSA. Fertilized embryos were assigned into sterile 25 mL glass beakers containing the designated concentration of ERM, 0.1% DMSO and 0.1, 1 and 10 µg/L PFOSA (n = 5 beakers of 10 fish each/treatment) in a 10 mL volume. The Supplemental Methods section contains full details on the analysis of ROS levels in zebrafish larvae.

### 2.5. Visual Motor Response Test

The visual motor response test was conducted following our published methods [24]. Fish were exposed continuously for 7 days with PFOSA as described above and assessed for locomotor activity behavior at a temperature of 27 ± 1 °C and a photoperiod of 14:10 h using the DanioVision instrument (Noldus, Wageningen, The Netherlands). The Supplemental Methods section contains full details on visual motor response tests.

### 2.6. Real-Time PCR

The Supplemental Methods section contains full details on real-time PCR assays. Briefly, zebrafish larvae at 6 hpf were exposed to either ERM, 0.1% DMSO or 0.1, 1, or 10 µg/L PFOSA. Each beaker contained 10–15 embryos and exposure conditions were maintained as mentioned above. Following the 7-day exposure period, larvae were pooled within a beaker, subjected to liquid nitrogen, and placed at −80 °C for RNA extraction. Sample sizes ranged from 4 to 6 beakers per group for gene expression analysis. The primers used in this study were obtained from published literature [25,26,27,28,29,30,31,32,33,34] and all targets measured in this study are listed in Supplemental Table S1. Two housekeeping genes (ribosomal subunit 18, *rps18*, and beta actin, *b*-actin) were used to normalize expression levels of all target genes. Normalized expression was calculated using CFX Manager™ software (v3.1) (baseline subtracted) and the Cq method.

### 2.7. Statistical Analysis

All data were compared to the solvent control (DMSO group). A log-rank test (Mantel–Cox) was employed to evaluate survival data. Data for hatch times were evaluated using a One-Way ANOVA at each time point. Levels of ROS and relative mRNA levels were first log(10) transformed following a Shapiro–Wilk test for normality. Group mean differences were then tested using a One-Way ANOVA (Dunnett’s multiple comparisons test). A simple linear regression was also conducted on the gene expression data to determine whether expression varied with concentration. Because there was no difference in expression between the ERM and DMSO group, these two experimental groups were combined for the regression as a “control” or baseline group. For the VMR, distance moved for larval fish in each treatment across the three independent experiments were binned into a single graph, but each individual run is shown in Supplemental Figure. The distance moved for fish in the DMSO group was normalized to a value of 1, and all treatments were compared relative to this group. A Kruskal–Wallis test followed by Dunn’s multiple comparisons test was employed to analyze discrete temporal units (light and dark periods) as the data were not normally distributed. All data are presented as mean ± S.D. Significance of difference was determined using a threshold of *p* < 0.05. Statistics and graphing were performed using GraphPad V9.3 (La Jolla, CA, USA).

## 3. Results

### 3.1. Survival, Hatch Rate, and Deformity

**Figure 1:**
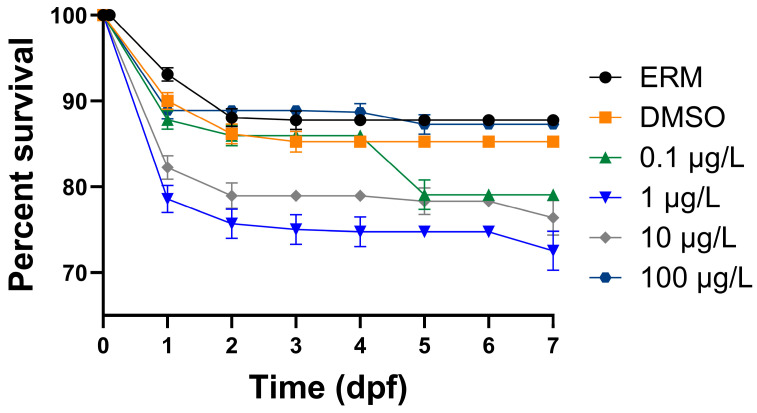
Percent of surviving zebrafish following exposure to one of either ERM, 0.1% DMSO or 0.1, 1, 10, or 100 µg/L PFOSA over 7 days. Error bars (S.E.M.) are small and within symbols in some cases.

**Figure 2:**
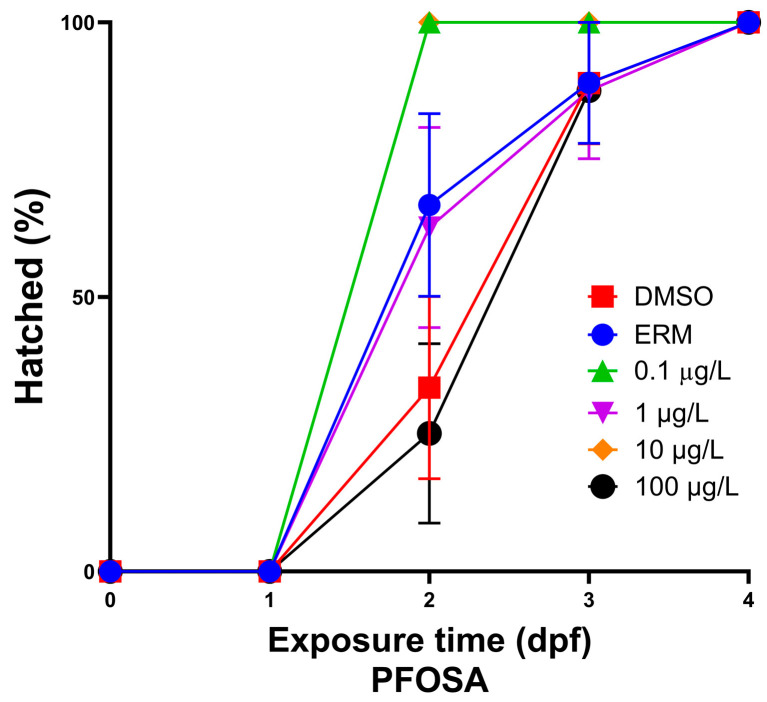
Total percent of hatched zebrafish embryos/larvae following exposure to one of either ERM, 0.1% DMSO or 0.1, 1, 10, or 100 µg/L PFOSA over 7 days.

After zebrafish were exposed to several concentrations of PFOSA for a period of 7 days, intriguingly only fish treated with 1 µg/L PFOSA exhibited significantly decreased survival [chi square = 88.68, df = 5, *p* value < 0.0001] (Figure 1) relative to the DMSO control group. Survival was approximately 10% lower for some eggs treated with PFOSA and these responses were most notable in the first 48 h of exposure. The hatch rate increased in fish treated with 0.1 and 10 µg/L PFOSA as all embryos were hatched at 2 dpf compared to the other treatment groups (*p* < 0.05) (Figure 2). There were no significant deformities observed; however, the few zebrafish that did have deformities (less than 3%) had caudal tail malformations.

### 3.2. Reactive Oxygen Species

**Figure 3:**
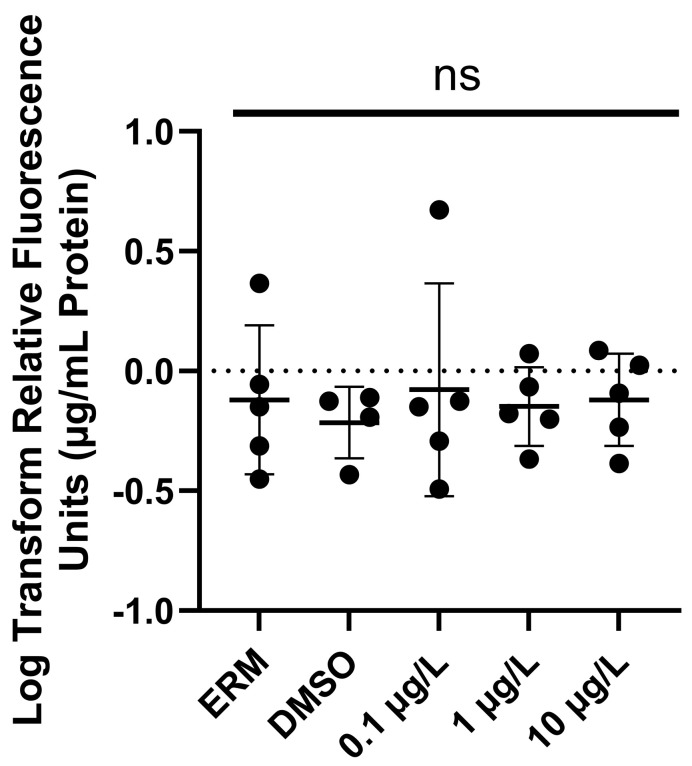
Normalized reactive oxygen species (to µg/mL media/protein). Each circle represents a biological replicate; the mean (±S.D.) is represented by the horizontal line (One-Way ANOVA and Dunnett’s multiple comparisons test; n = 5/treatment). ns = not significant.

We assessed the effects of PFOSA on ROS induction in larval zebrafish at 7 dpf. PFOSA exposure did not significantly affect ROS levels (F _(4, 19)_ = 0.14, *p* = 0.96) (Figure 3).

### 3.3. Visual Motor Response Test

**Figure 4:**
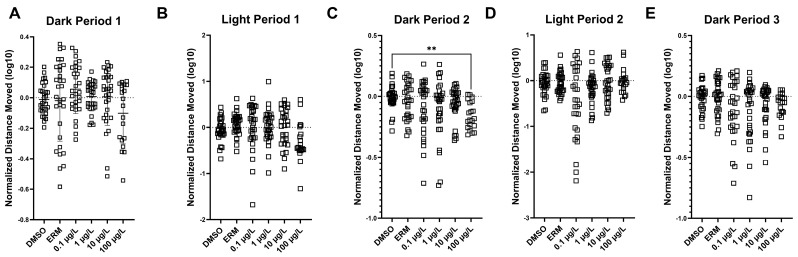
The distance moved in each of the light and dark zones (10 min bins) of 7-day zebrafish larvae exposed to 0.1% DMSO, ERM or 0.1, 1, 10, or 100 µg/L PFOSA. Graphs are the combined output from three independent VMR runs. Columns depict mean (±S.D.) (Kruskal–Wallis test and Dunn’s multiple comparisons test; n = 8–12 fish/treatment/run). Asterisk indicates difference at ** *p* < 0.01.

Three independent trials were conducted, and data for the relative distance travelled (log transformed and relative to the DMSO control standardized to a value of 1) were combined for each experiment. Zebrafish larvae exposed to 100 µg/L exhibited reduced activity in Dark Period 2 only (number of groups = 6, Kruskal–Wallis statistic = 13.60; *p* value = 0.018) (Figure 4, panel C). Individual VMR trials can be found in Supplementary Figure, Supplemental Figure S1.

### 3.4. Expression Analysis of Transcripts

**Figure 5:**
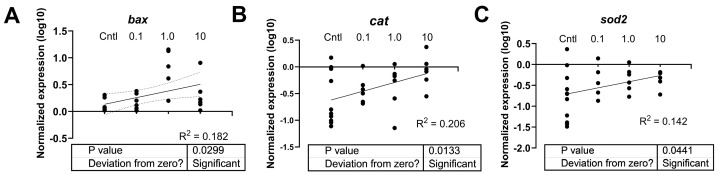
Linear regression for relative expression of (**A**) *bax*, (**B**) *cat,* and (**C**) *sod2* in 7-day old zebrafish. The DMSO and ERM group were combined (“Cntl”). Each circle indicates a biological replicate or beaker of pooled fish (n = 4 to 6). The solid line indicates the relationship between expression and the region between the two outermost dotted lines is the 95% confidence interval of the X intercept.

**Figure 6:**
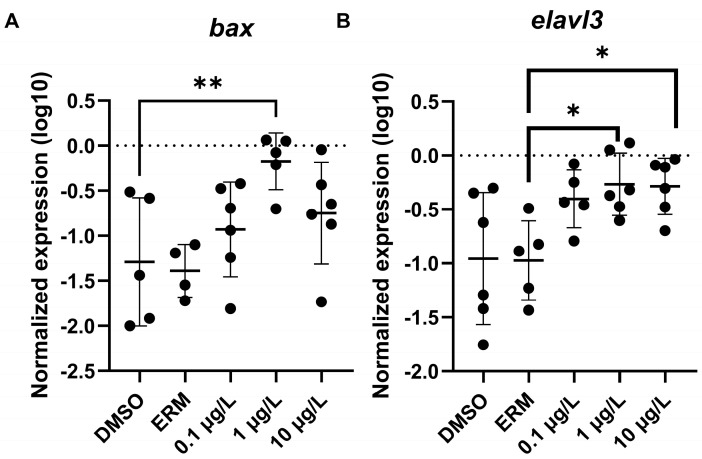
Relative expression of (**A**) *bax* and (**B**) *elavl3* in 7-day old larval zebrafish exposed to 0.1% DMSO, ERM or 0.1, 1, or 10 µg/L PFOSA. Each circle is a beaker of pooled fish (biological replicate), and the mean (±S.D.) is indicated by the horizontal line (One-Way ANOVA, Dunnett’s multiple comparisons test; n = 4 to 6). Asterisk indicates * *p* < 0.05 or ** *p* < 0.01.

The effects of PFOSA on mRNA steady state levels were measured in larval fish. Regression analysis revealed that the apoptosis-related transcript (*bax*) on oxidative stress-related transcripts (*cat*, *sod2*) increased with increasing concentrations of PFOSA and the statistical information and R^2^ of the linear regressions are shown in Figure 5. Bax mRNA levels were elevated in zebrafish from the 1 µg/L PFOSA treatment compared to the DMSO control group (F _(4, 21)_ = 4.160, *p* = 0.0123) (Figure 6A).

**Figure 7:**
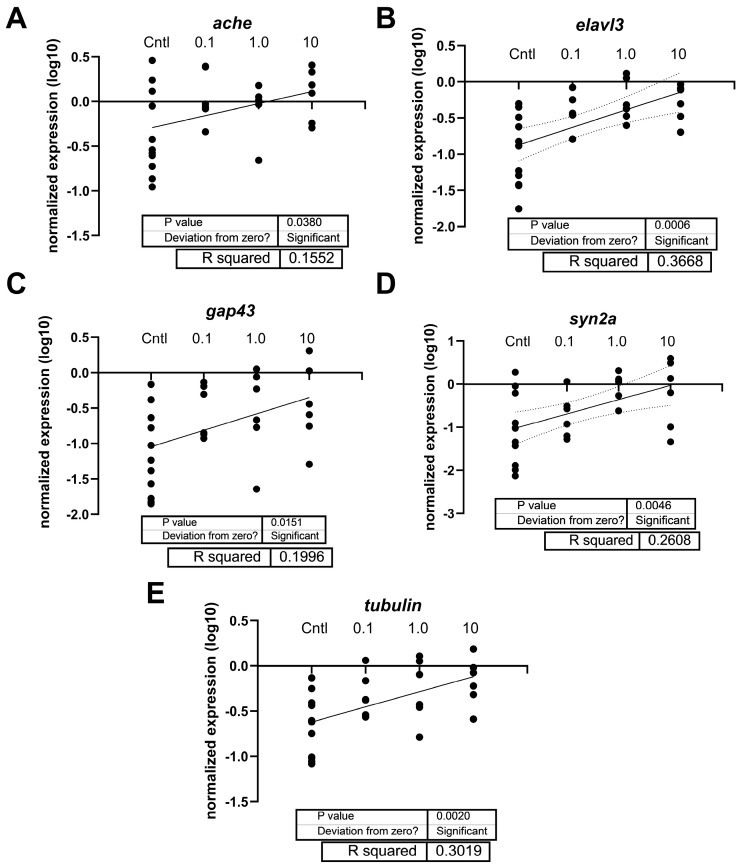
Linear regression for relative expression of (**A**) *ache*, (**B**) *elavl3*, (**C**) *gap43*, (**D**) *syn2a*, and (**E**) *tubulin (tubb3)* in 7-day old zebrafish. The DMSO and ERM group were combined (“Cntl”). Each circle indicates a biological replicate or beaker of pooled fish (n = 4 to 6). The solid line indicates the relationship between expression and the region between the two outermost dotted lines is the 95% confidence interval of the X intercept.

Regarding neurotoxicity-related genes, transcript levels of *elavl3* were significantly elevated in zebrafish treated with 1 and 10 µg/L PFOSA (F _(4, 23)_ = 4.802, *p* = 0.0058) (Figure 6B). No other transcript was significantly different when comparing group means to the mean of the DMSO control (*p* > 0.05); however, regression analysis revealed that many neurotoxicity transcripts showed a concentration independent response with PFOSA, and several transcripts were increased with higher exposure concentrations (Figure 7). The statistical information and R^2^ of the simple linear regressions are provided in the figures.

## 4. Discussion

We observed that PFOSA affected the survival of zebrafish treated with some concentrations of PFOSA 1 µg/L (10% decline at this concentration relative to the DMSO control). Dasgupta et al. (2020) exposed zebrafish embryos to 0.78–50 µM (389–24,900 µg/L) PFOSA for up to 72 hpf and 100% mortality was observed in all treated embryos by 72 hpf [18]. Regarding abnormalities, we observed only a few deformities (less than 2–3 percent) across all treatment groups; several studies examining toxicity effects of PFOSA report significant deformities, which are likely due to much higher treatment concentrations. It was also reported that all treated embryos exhibiting concentration- and duration-dependent abnormalities, as well as developmental delays, at 24 hpf. For instance, embryos exposed to PFOSA starting at 0.75 hpf exhibited stronger concentration-dependent delays in epiboly compared to embryos exposed at 4 or 5 hpf. Truong et al. (2022) exposed dechorionated zebrafish embryos to 0.015–100 µM (7.4–49,900 µg/L) of various PFAS, including PFOSA from 6 to 120 hpf [35]. PFAS were ranked on potency based on morphological effects (i.e., pericardial and yolk sac edema, brain and eye malformation) in which PFOSA was ranked second highest. Various studies also report cardiac abnormalities, including heart elongation and reduced cardiac output, heart rate, and stroke volume, in zebrafish exposed to concentrations in the range of 0.01–100 µg/L [16,17]. In our case with survival, 1 µg/L may have been too low a concentration to sufficiently activate defense mechanisms to PFOSA exposure, leading to lower overall survival in developing fish while higher concentrations of PFOSA may elicit a stronger defense response to mitigate toxicity, leading to higher survival. Such dose-dependent responses have been observed for other chemical exposures in zebrafish [36,37]. This hypothesis is supported by the increase in anti-oxidant defense enzymes with higher concentrations of PFOSA. Nevertheless, survival remained relatively high (80–90%) for fish exposed up to 100 µg/L PFOSA, suggesting that the chemical, spanning environmental concentrations, is not overtly toxic up to 100 µg/L.

The amount of ROS is often indicative of the amount of oxidative stress in cells, and an excess of ROS can contribute to damage at the molecular level. Limited studies in the literature examine the impact PFOSA has on organisms. In our study, we did not observe any increase in ROS in zebrafish treated with PFOSA; however, there was a concentration-dependent increase in both *cat* and *sod2* expression. Mitochondrial dysfunction has been thought to contribute to the progression of neurodegenerative disorders and the presence of ROS is one clear indicator of dysfunction as antioxidant systems are implemented to counteract oxidative stress. This supports our oxidative stress-related gene responses (PFOSA-induced increase in *cat* and *sod2* mRNA levels), which could have mitigated any change in ROS levels in the larval zebrafish. Similar results have also been observed in rodent models where oxidative stress-related genes, like *cat*, were significantly increased to counteract damage by PFOA-induced lipid peroxidation in mouse brain and liver tissues [38]. ROS can also trigger apoptosis to mediate inflammation. Though PFOSA was found to only significantly upregulate two apoptosis-related genes (*bax* and *cycs*) in our study, another study reports increased apoptotic cells in the brain and upregulated *bcl-2*, *caspase3*, and *p53* zebrafish exposed to PFOS [39]. Bax is a pro-apoptotic factor in the Bcl-2 family, signaling mitochondria and cell death, while cytochrome c is an intrinsic apoptotic signal activating downstream caspase enzymes. Other studies investigating PFOSA report mixed results for antioxidant gene expression and proteins. Olufsen and Arukwe (2015) exposed Atlantic salmon (*Salmo salar*) hepatocytes to 25 or 50 µM (12,400–24,900 µg/L) PFOSA for 24 or 48 h and analyzed catalase, glutathione peroxidase, glucocorticoid receptor, and glutathione S-transferase mRNA levels, which were not significantly impacted [40]. Another study also exposed Atlantic salmon hepatocytes to 2, 20, or 50 µM (998, 9900, or 24,900 µg/L) PFOSA for 12 or 24 h [3]. No significant changes to *gpx* mRNA levels were found, but *cat* mRNA levels were significantly increased by 20 and 50 µM PFOSA following 24 h of exposure, suggesting that antioxidant defense mechanisms were activated. Differences among studies may occur due to the type of model used to investigate PFOSA toxicity (e.g., cells versus larvae). Taken together, there is evidence that PFOSA initiates an antioxidant defense and any elevation in ROS may lead to higher levels of apoptosis in larval zebrafish.

We hypothesized that PFOSA would induce neurotoxicity in the form of behavioral changes and altered expression of genes related to neurotoxicity. Indeed, we observed hypoactivity at 100 µg/L, which corresponded to the highest expression levels of several neurotoxicity biomarkers. These responses indicate some form of neurological impairment [41]. Chemical neurotoxins cause damage to, or death of, cells in the nervous system, disrupting neuronal pathways linked to neurodegenerative illnesses and other neurodevelopmental issues (i.e., Parkinson’s disease and schizophrenia). For instance, zebrafish exposed to PFOS had transcriptome changes linked to disturbance of the neuromuscular system [42] and zebrafish exposed to perfluorononanoic acid (PFNA) showed evidence of altered neuroinflammatory pathways [43]. To our knowledge, this is the first study to examine the neurotoxicity mechanism in zebrafish exposed to PFOSA. We observed hypoactivity effects on locomotor activity in larval zebrafish, suggesting neurotoxicity or motor deficits with PFOSA exposure. Fish treated with 100 µg/L PFOSA showed reduced activity in Dark Period 2 of the combined VMR. Truong et al. (2022) exposed dechorionated zebrafish embryos to 0.015–100 µM PFOSA from 6 to 120 hpf and found that PFOSA induced both a refractory and an excitatory phase of hyperactivity [35]. Consistent with our observation, after exposing zebrafish embryos to 1 or 10 µg/L PFOSA for 120 h, Liu et al. (2022) observed reductions in the total distance moved, average swimming velocity, and maximum acceleration in fish treated with 1 µg/L PFOSA [17]. Our results also revealed that PFOSA alters the expression of neurotoxicity-related genes, as notable effects were observed in *elavl3*, and positive associations were detected between PFOSA concentration and expression levels (e.g., *ache*, *elavl3*, *gap43*, *syn2a*, and *tubb3*). *Elavl3* is expressed in different nervous system structures and is known to regulate neurogenesis [44]. Additionally, *ache* is involved in cholinergic functioning and *syn2a* is involved in dopamine and serotonin release. PFAS exposure has previously been shown to alter these transcripts; PFOS, the metabolic product of PFOSA, was reported to decrease *ache* expression [45] and perfluorododecanoate (PFDoA) decreased mRNA levels of *elavl3*, *gap43*, and *syn2a* [46]. Here, we report an elevation in the expression of *elavl3* and many other neurotoxic-related transcripts with PFOSA exposure, and this may reflect a compensatory response to impaired neurogenesis and neurotransmitter release. Conversely, different types of PFAS may elicit unique responses in the CNS in relation to gene expression patterns. Regardless, there is evidence from the molecular response that PFOSA alters genes related to neuronal integrity and structure, suggesting the potential for neurotoxicity in developing larval fish. Thus, early developmental exposures to PFOSA may have long-lasting detrimental effects on the adult brain and this should be further investigated.

## 5. Conclusions

In summary, very few studies have been carried out on PFOSA and there is little information on PFOSA’s environmental presence and accumulation in aquatic species. PFOSA did not significantly impact the prevalence of malformations or reactive oxygen species generated in larval fish; however, PFOSA did affect locomotor activity and transcripts related to oxidative damage response, apoptosis, and neurotoxicity. Further mechanistic studies in zebrafish are warranted to further address PFOSA neurotoxicity in the CNS. This study contributes to our knowledge regarding the relative toxicity of PFAS on fish to assist future risk assessment methodologies of these concerning, persistent environmental pollutants.

## Data Availability

Data will be made available upon request.

## Supplementary Materials

The following supporting information can be downloaded at https://www.mdpi.com/article/10.3390/toxics12100723/s1: Figure S1: Independent VMR experiments; Table S1: Primers used for qPCR analysis.
