# Supplementary material for: Adverse Outcomes Following Exposure to Perfluorooctanesulfonamide (PFOSA) in Larval Zebrafish (Danio rerio): A Neurotoxic and Behavioral Perspective
Source: Toxics. 2024 Oct 4;12(10):723. doi: 10.3390/toxics12100723 (PMC11510739; doi:10.3390/toxics12100723)
Supplement: Supplementary file 1 [file toxics-12-00723-s001.zip › toxics-3187897-supplementary.pdf]

Supplemental Methods and Results

Adverse Outcomes Following Exposure to Perfluorooctanesulfonamide (PFOSA) in Larval Zebrafish (*Danio rerio*): A Neurotoxic and Behavioral Perspective

Nikita David[1], Emma Ivantsova[1], Isaac Konig[1,2], Cole English[1], Lev Avidan[1], Mark Kreychman[1], Mario Rivera[1], Camilo Escobar [1], Eliana Maira Agostini Valle[1,3], Amany Sultan[1,4], Christopher J. Martyniuk[1,5]*

1 Center for Environmental and Human Toxicology, Department of Physiological Sciences, College of Veterinary Medicine, University of Florida, Gainesville, Florida, 32611, USA

2 Department of Chemistry, Federal University of Lavras (UFLA), Minas Gerais, Brazil

3 Universidade Federal de São Paulo – Instituto de Ciencias Ambientais, Quimicas e Farmaceuticas – Campus Diadema – Brazil

4 Animal Health Research Institute, Agriculture Research Centre, Giza, Egypt.

5 UF Genetics Institute, Interdisciplinary Program in Biomedical Sciences Neuroscience, University of Florida

* Correspondence: Chris Martyniuk, email: cmartyn@ufl.edu; ORCID: 0000-0003-0921-4796

Supplemental Methods

2.1. Chemical Preparation

Perfluorooctanesulfonamide (PFOSA, (1,1,2,2,3,3,4,4,5,5,6,6,7,7,8,8,8-heptadecafluorooctane-1-sulfonamide Perfluorooctane sulfonamide) (CAS Number: 754-91-6, purity > 95%) was purchased from Fisher Scientific (Cat# AC459640010). Stock solutions of PFOSA were prepared in dimethyl sulfoxide (DMSO, dimethyl sulfoxide, CAS 67-68-5, Sigma Aldrich) and added to embryo rearing media (ERM) containing the zebrafish embryos. Recipes for ERM can be found in [20]. Exposure solutions were prepared daily to yield final nominal environmentally relevant concentrations of 0.1, 1, 10, and 100 µg/L PFOSA with a final concentration < 0.1% v/v DMSO in experimental treatments.

2.2. Maintenance and Egg Production of Zebrafish

Adult zebrafish (AB x Tübingen, Danio rerio) were raised in a flow-through Pentair system in the Cancer-Genetics Research Center at the University of Florida as outlined previously [21,22]. The University of Florida maintains a breeding colony for research and outbreeds extraneous fish to maintain high genetic diversity. Rearing and staging of zebrafish embryos followed that described by [23]. Adult fish were fed Zeigler Zebrafish Diet ad libitum. Zebrafish at 6 months of age were selected at random from a breeding stock and placed into a shallow water breeding tank the night before embryo collection (2 males and 2 females). A divider was used to separate the males and females overnight and it was removed at 8:00 am when the facility lights turned on. Typically, three or four breeding tanks are prepared the night before to maximize eggs, and adult fish are bred once every 1 to 2 weeks. Embryos were rinsed with saline phosphate buffer

(PBS) and ERM several times once they were collected and they were sorted under a light microscope in the late morning (around 4 hours post-fertilization, hpf). All experiments were conducted at pH = 7.2 ± 0.5, a conductivity value of 600 ± 100 µS/cm, light/dark cycle of 14:10 h, dissolved oxygen concentration ~80% air saturation, and temperature of 27 ± 1 °C. The Institutional Animal Care and Use Committee of University of Florida approved all the experiments (UFIACUC202300000140).

2.3. PFOSA Exposure Regime

Fertilized and normally developing eggs were selected at ∼6 h post-fertilization (hpf) using a dissecting microscope. Zebrafish eggs were assigned in random fashion into experimental groups [ERM, 0.1% DMSO, or one dose of 0.1, 1, 10, 100 µg/L PFOSA]. Four independent experiments were conducted using embryos that were generated from separate breeders of fish. For each experiment, there were 5 to 6 replicate glass beakers for each experimental group containing 20-30 embryos and 10 mL of embryo rearing media (ERM). Following the addition of chemicals to the water, the glass beakers were placed into an incubator that was maintained at 27 ± 1 °C. Each day, data related to mortality, deformities, hatch times and images using an EVOS™ FL Auto Imaging System (ThermoFisher Scientific, USA) were collected. Deformity assessments included the presence of spinal lordosis and edema (yolk sack/pericardial) was noted over the duration of the exposure. Exposure solutions were prepared fresh daily from stock stored at -20°C in glass amber vials and water was renewed every day with a 90% water change.

2.4. Reactive Oxygen Species

Six hpf embryos were obtained immediately after fertilization and treated as per above for 7 days in the ERM with designated concentrations of PFOSA. Embryos were rinsed three times in ERM and assessed under a light microscope. Fertilized embryos were evenly distributed using sterile micropipettes into sterile 25 mL glass beakers containing the assigned concentration of ERM, 0.1% DMSO and 0.1, 1 or 10 µg/L PFOSA (n=5 beakers of 10 fish each/treatment) in a 10 mL volume. Embryos were maintained in the controlled environment of an incubator at 27 ± 1.0°C. Media changes were conducted every 24 hours with new sterile ERM or renewed PFOSA in ERM. Following a 7-day exposure, larvae were quickly transferred from beakers into 1.7 mL microcentrifuge tubes, homogenized in 200 µL of ice-cold PBS, and centrifuged at 12,000 g for 20 min at 4° C. Following centrifugation, 20 µL of the supernatant was transferred to a black fluorescence 96-well plate and incubated for 5 minutes at room temperature. After incubation, 8.3 µL of 1 mg/mL 2′,7′-Dichlorofluorescin Diacetate (Calbiochem, Millipore Sigma, CAS 4091–99, or H2-DCFDA) dissolved in DMSO and 200 µL of PBS were added to each well. Then, the plate was incubated in the dark for 30 minutes at 37 ± 1.0 °C. Following this, excitation at 485 nm and emission at 520 nm were recorded using a Synergy™ H4 Hybrid Multi-Mode Microplate Reader. Total protein using a BCA assay (Thermo Sci-entific) was measured to express ROS as normalized signal intensity/(µg/mL) protein.

2.5. Visual Motor Response Test

Experiments were performed to test the dark photokinesis response in larvae. Fish were exposed continuously for 7 days with 90% daily water changes with PFOSA as described above and assessed for locomotor activity behavior at a temperature of 27 ± 1 °C and photoperiod pf 14:10 h. In each trial, zebrafish embryos at 6 hpf were randomly assigned to an experimental group of

either ERM, 0.1% DMSO, or 0.1, 1, 10, or 100 µg/L PFOSA (5 beakers per treatment). Each group contained 20 zebrafish embryos and 10 mL of ERM. In mid-afternoon on the 7th day, 2 normally developed larva were selected from each replicate beaker and placed in a random fashion into a clear 96-well plate (N=10 fish per treatment/per experiment). Each well contained 200 µL of ERM. The 96-well plate was placed into DanioVision™ Observation Chamber (Noldus Information Technology, Leesburg, VA) with an infrared analog camera (25 frames/second) to track the activities of zebrafish larvae. The assay proceeded as per our previous methods [24]. Data were analyzed independently for each trial, and the total distance moved was used as an indicator of overall locomotor activity. Data were also analyzed by normalizing each of the three independent runs using a relative value = 1 for the solvent control group, and then relative data were combined into a single graph representing 30 larvae per treatment.

2.6. Real-Time PCR

Zebrafish larvae at 6 hpf were exposed to either ERM, 0.1% DMSO or 0.1, 1, or 10 µg/L PFOSA. Each beaker contained 10-15 embryos and exposure conditions were maintained as that above. Following the 7-day exposure period, larvae were pooled within a beaker, subjected to liquid nitrogen, and placed at −80 °C for RNA extraction. Extraction of RNA from larvae pools was performed using 500 mL TRIzol® Reagent (Life Tech-nologies, Carlsbad, CA, USA) as per the manufacturer's protocol. Samples were DNase treated with DNA TURBO (Ambion). DNase-treated samples were assessed for quality using the 2100 Bioanalyzer (Agilent Technologies, Santa Clara, CA, USA). The mean RIN value for RNA was >7 and concentrations were approximately 50-200 ng/µl sample. The cDNA synthesis was performed using ~500 ng of column-purified RNA using iS-cript (BioRad) following the manufacturer's protocol in a final

sample volume of 15 µL. Once prepared, samples were placed into a T100™ Thermal Cycler (BioRad, USA). The cDNA was generated using the following steps: 25 °C for 5 min, 42 °C for 30 min, 85 °C for 5 min, and 4 °C for 5 min. Prior to real-time PCR, cDNA stocks were diluted 1:25 in RNAse-DNase free water. The no reverse transcriptase (NRT) controls were prepared in the same way as above without enzyme using 3 randomly selected RNA samples.

Real-time PCR was performed using the CFX Connect™ Real-Time PCR Detection System (BioRad) with SsoFast™ EvaGreen® Supermix (BioRad, Hercules, CA, USA), 200-300 nM of each forward and reverse primer, and 3.33 µL of diluted cDNA. The two-step thermal cycling parameters were as follows: initial 1-cycle Taq polymerase activation at 95 °C for 30 s, followed by 95 °C for 5 s, and 60 °C primer annealing temperature for 5 s. After 40 cycles, a dissociation curve was generated, starting at 65.0 and ending at 95.0°C, with increments of 0.5 °C every 5 s. Primers used in this study were obtained from published literature [25-34] (Supplemental Table S1). Two housekeeping genes (ribosomal subunit 18, rps18, and beta actin, b-actin) were used to normalize expression levels of all target genes. Target genes included acetylcholinesterase (ache), BCL2-Associated X Apoptosis Regulator (bax), BCL2 Apoptosis Regulator (bcl2), catalase (cat), caspase 3 (caspase3), ELAV-like RNA binding protein 3 (elavl3), growth-asso-ciated protein 43 (gap43), glial fibrillary acidic protein (gfap), heat shock protein 70 (hsp70), mesencephalic astrocyte derived neurotrophic factor (manf), myelin basic protein (mbp), nestin (nestin), tumor protein 53 (p53), sonic hedgehog signaling molecule (shha), superoxide dismutase 1 (sod1) (Cu/Zn SOD), superoxide dismutase 2 (sod2) (Mn SOD), synapsin IIa (syn2a), and tubulin. Normalized expression was obtained for each target gene using CFX Manager™ software (v3.1) (baseline subtracted) and the Cq method was employed. The qPCR analysis included 3 NRT samples and 1 NTC sample. Negative controls indicated that RNA

column purification and DNase treatment sufficiently removed gDNA. Sample sizes ranged from 4 to 6 for gene expression analysis. All primers used in the qPCR analysis amplified one product, indicated by a single melt curve.

2.7. Statistical analysis

All data were compared to the solvent control (DMSO group). A log-rank test (Mantel–Cox) was employed to evaluate survival data. Data for hatch times were evaluated using a One-Way ANOVA at each time point. Levels of ROS and relative mRNA levels were first log(10) transformed following a Shapiro–Wilk test for normality. Group mean differences were then tested using a One-Way ANOVA (Dunnett's multiple comparisons test). A simple linear regression was also conducted on the gene expression data to determine whether expression varied with concentration. Because there was no difference in expression between the ERM and DMSO group, these two experimental groups were combined for the regression as a "control" or baseline group. For the VMR, the distances moved for larval fish in each treatment across the three independent experiments were binned into a single graph, but each individual run is shown in the Supplemental Figures. The distance moved in the DMSO group was normalized to a value of 1, and all treatments were compared relative to this group. A Kruskal–Wallis test followed by a Dunn's multiple comparisons test was used to evaluate differences in locomotor activity, which was analyzed as discrete temporal units (light and dark sections to corresponding control group) as the data were not normally distributed. Data are presented as mean ± S.D. Significance of difference was determined using a threshold of $P < 0.05$. Statistics and graphing were performed using GraphPad V9.3 (La Jolla, CA, USA).

Supplemental Results

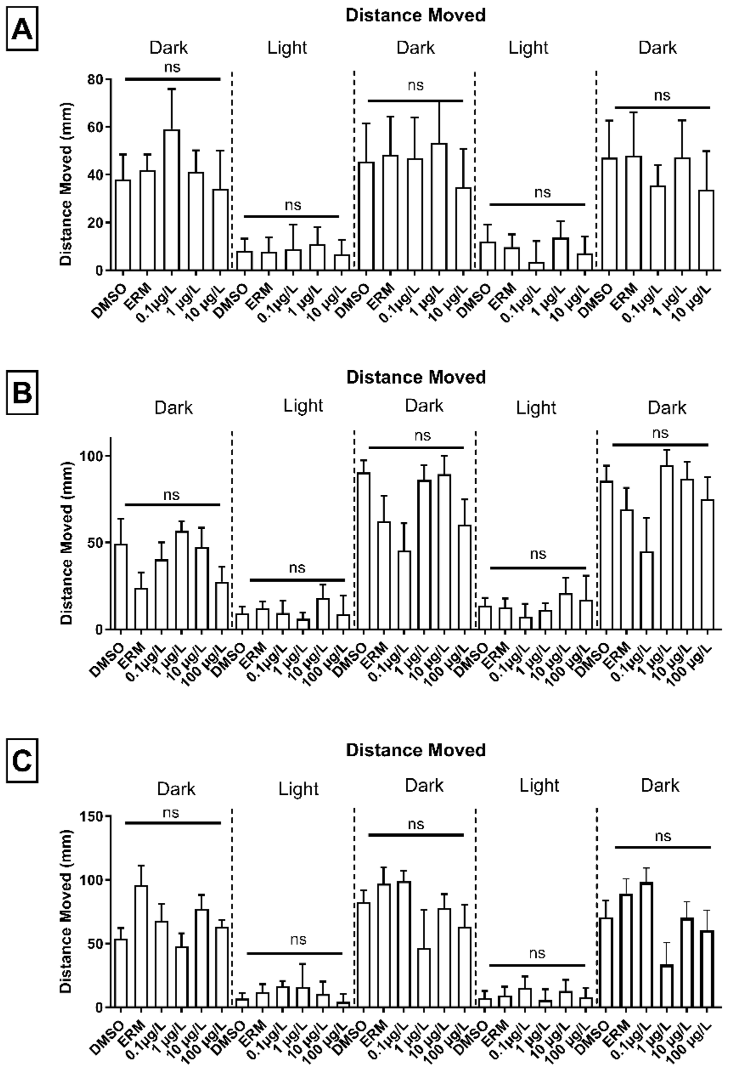

Supplemental Figure S1. The distance moved in each of the light and dark zones (10-minute bins) of 7-day zebrafish larvae exposed to 0.1% DMSO, ERM and 0.1, 1, 10, or 100 µg/L PFOSA in 3 separate experiments. Mean values are depicted by the columns in each dark–light phase (mean ± S.D.) (N=8-12 fish/treatment/experiment).

Supplemental Tables:

Supplemental Table S1. Primers used for real-time PCR analysis.

Gene Name	Gene Symbol	Forward (5' to 3')	Reverse (5' to 3')	Reference
Acetylcholinesterase	*ache*	GCTAATGAGCAAAAGCATGTGGGC	TATCTGTGATGTTAAGCAGACGAGGCA	NM_131846.2
Beta-actin	*bactin*	CGAGCAGGAGATGGGAACC	CAACGGAAACGCTCATTGC	Wang et al. 2018
BCL2-Associated X Apoptosis Regulator	*bax*	GACTTGGGAGCTGCACTTCT	TCCGATCTGCTGCAAACACT	Fang et al., 2024
BCL2 Apoptosis Regulator	*bcl2*	AGGAAAATGGAGGTTGGGATG	TGTTAGGTATGAAAACGGGTGGA	Fang et al., 2024
Catalase	*cat*	CAAGGTCTGGTCCCATAAA	TGACTGGTAGTTGGAGGTAA	Fang et al., 2024
Caspase 3	*caspase3*	CCGCTGCCCATCACTA	ATCCTTTCACGACCATCT	Fang et al., 2024
ELAV-Like RNA Binding Protein 3	*elavl3*	AGACAAGATCACAGGCCAGAGCTT	TGGTCTGCAGTTTGAGACCGTTGA	Yang et al. 2023
Growth-Associated Protein 43	*gap43*	TTAACGGAGGACCAGTGCAA	GTCCTGATCTCCAGCACACG	Dong et al., 2023
Glial Fibrillary Acidic Protein	*gfap*	GGATGCAGCCAATCGTAAT	TTCCAGGTCACAGGTCAG	Dong et al., 2023
Heat Shock Protein 70	*hsp70*	GAAGACGGCATCTTTGAGGTGA	GGGCCCTCTTGTTCTGACTGAT	Hahn et al., 2014
Mesencephalic Astrocyte-Derived Neurotrophic Factor	*manf*	AGATGGAGAGTGTGAAGTCTGTGTG	CAATTGAGTCGCTGTCAAAACTTG	Yang et al., 2023
Myelin Basic Protein	*mbp*	AATCAGCAGGTTCTTCGGAGGAGA	AAGAAATGCACGACAGGGTTGACG	Yang et al., 2023
Nestin	*nestin*	ATGCTGGAGAAACATGCCATGCAG	AGGGTGTTTACTTGGGCCTGAAGA	Jiang et al., 2018

Tumor Protein P53	*p53*	CCCGGATGGAGATAACTTG	CACAGTTGTCCATTCAGCAC	Fang et al., 2024
Ribosomal 18s	*rps18*	TCGCTAGTTGGCATCGTTTATG	CGGAGGTTCGAAGACGATCA	McCurley and Callard, 2008
Sonic Hedgehog Signaling Molecule	*shha*	AGACCGAGACTCCACGACGC	TGCAGTCACTGGTGCGAACG	Guo et al., 2023
Superoxide dismutase 1	*sod1 (Cu/Zn SOD)*	CAACACAAACGGCTGCATCA	TTTGCAACACCACTGGCATC	Sarkar et al., 2014
Superoxide dismutase 2	*sod2 (Mn SOD)*	AGCGTGACTTTGGCTCATTT	ATGAGACCTGTGGTCCCTTG	Sarkar et al., 2014
Synapsin IIa	*syn2a*	GTACCATGCCAGCATTTC	TGGTTCTCCACTTTCACCTT	Guo et al., 2023
Tubulin 3	*tubulin*	AATCACCAATGCTTGCTTCGAGCC	TTCACGTCTTTGGGTACCACGT CA	Wu et al., 2016

Dong, M., Wang, J., Liu, Y., He, Q., Sun, H., Xu, Z., ... & Gao, P. (2023). 3-Bromocarbazole-Induced Developmental Neurotoxicity and Effect Mechanisms in Zebrafish. *ACS ES&T Water*.

Fang, C., Di, S., Yu, Y., Qi, P., Wang, X., & Jin, Y. (2024). 6PPD induced cardiac dysfunction in zebrafish associated with mitochondrial damage and inhibition of autophagy processes. *Journal of hazardous materials*, *471*, 134357. https://doi.org/10.1016/j.jhazmat.2024.134357

Guo, Y., Fu, Y., & Sun, W. (2023). 50 Hz Magnetic Field Exposure Inhibited Spontaneous Movement of Zebrafish Larvae through ROS-Mediated syn2a Expression. *International journal of molecular sciences*, *24*(8), 7576. https://doi.org/10.3390/ijms24087576.

Hahn, M.E., McArthur, A.G., Karchner, S.I., Franks, D.G., Jenny, M.J., Timme-Laragy, A.R., Stegeman, J.J., Woodin, B.R., Cipriano, M.J. and Linney, E., 2014. The transcriptional response to oxidative stress during vertebrate development: effects of tert-butylhydroquinone and 2, 3, 7, 8-tetrachlorodibenzo-p-dioxin. PloS one, 9(11), p.e113158.

Jiang, F., Liu, J., Zeng, X., Yu, L., Liu, C. and Wang, J., 2018. Tris (2-butoxyethyl) phosphate affects motor behavior and axonal growth in zebrafish (Danio rerio) larvae. *Aquatic Toxicology*, *198*, pp.215-223.

McCurley AT, Callard GV. Characterization of housekeeping genes in zebrafish: male-female differences and effects of tissue type, developmental stage and chemical treatment. BMC Mol Biol. 2008 Nov 12;9:102. doi: 10.1186/1471-2199-9-102. PMID: 19014500; PMCID: PMC2588455.

Sarkar, S., Mukherjee, S., Chattopadhyay, A. and Bhattacharya, S., 2014. Low dose of arsenic trioxide triggers oxidative stress in zebrafish brain: expression of antioxidant genes. Ecotoxicology and environmental safety, 107, pp.1-8.

Wang, X.H., Souders 2nd, C.L., Zhao, Y.H., Martyniuk, C.J. 2018. Paraquat affects mitochondrial bioenergetics, dopamine system expression, and locomotor activity in zebrafish (Danio rerio). Chemosphere. 191, 106-117.

Wu, Q., Yan, W., Liu, C., Li, L., Yu, L., Zhao, S. and Li, G., 2016. Microcystin-LR exposure induces developmental neurotoxicity in zebrafish embryo. Environmental Pollution, 213, pp.793-800.

Yang Q, Deng P, Xing D, Liu H, Shi F, Hu L, Zou X, Nie H, Zuo J, Zhuang Z, Pan M, Chen J, Li G. Developmental Neurotoxicity of Difenoconazole in Zebrafish Embryos. Toxics. 2023 Apr 8;11(4):353. doi: 10.3390/toxics11040353. PMID: 37112580; PMCID: PMC10142703.
